# Nasal and Pharyngeal Mucosal Immunity to Poliovirus in Children Following Routine Immunization With Inactivated Polio Vaccine in the United States

**DOI:** 10.1093/infdis/jiae264

**Published:** 2024-05-29

**Authors:** Audrey Godin, Ruth I Connor, Hanna N Degefu, Pamela C Rosato, Wendy F Wieland-Alter, Katherine S Axelrod, Gabriela Kovacikova, Joshua A Weiner, Margaret E Ackerman, Eunice Y Chen, Minetaro Arita, Ananda S Bandyopadhyay, Amber I Raja, John F Modlin, Elizabeth B Brickley, Peter F Wright

**Affiliations:** Health Equity Action Lab, Department of Infectious Disease Epidemiology and International Health, London School of Hygiene & Tropical Medicine, London, United Kingdom; Department of Pediatrics, Geisel School of Medicine at Dartmouth, Dartmouth Health, Lebanon, New Hampshire, USA; Department of Microbiology and Immunology, Geisel School of Medicine at Dartmouth, Hanover, New Hampshire, USA; Department of Microbiology and Immunology, Geisel School of Medicine at Dartmouth, Hanover, New Hampshire, USA; Department of Pediatrics, Geisel School of Medicine at Dartmouth, Dartmouth Health, Lebanon, New Hampshire, USA; Thayer School of Engineering, Dartmouth College, Hanover, New Hampshire, USA; Thayer School of Engineering, Dartmouth College, Hanover, New Hampshire, USA; Thayer School of Engineering, Dartmouth College, Hanover, New Hampshire, USA; Department of Microbiology and Immunology, Geisel School of Medicine at Dartmouth, Hanover, New Hampshire, USA; Thayer School of Engineering, Dartmouth College, Hanover, New Hampshire, USA; Department of Pediatrics, Geisel School of Medicine at Dartmouth, Dartmouth Health, Lebanon, New Hampshire, USA; Department of Surgery, Geisel School of Medicine at Dartmouth, Dartmouth Health, Lebanon, New Hampshire, USA; Department of Virology II, National Institute of Infectious Diseases, Tokyo, Japan; Polio, Global Development, Bill & Melinda Gates Foundation, Seattle, Washington, USA; Health Equity Action Lab, Department of Infectious Disease Epidemiology and International Health, London School of Hygiene & Tropical Medicine, London, United Kingdom; Department of Pediatrics, Geisel School of Medicine at Dartmouth, Dartmouth Health, Lebanon, New Hampshire, USA; Health Equity Action Lab, Department of Infectious Disease Epidemiology and International Health, London School of Hygiene & Tropical Medicine, London, United Kingdom; Department of Pediatrics, Geisel School of Medicine at Dartmouth, Dartmouth Health, Lebanon, New Hampshire, USA

**Keywords:** poliovirus, vaccines, mucosal immunity, nasal cavity, pharynx

## Abstract

**Background:**

Although polioviruses (PVs) replicate in lymphoid tissue of both the pharynx and ileum, research on polio vaccine–induced mucosal immunity has predominantly focused on intestinal neutralizing and binding antibody levels measured in stool.

**Methods:**

To investigate the extent to which routine immunization with intramuscularly injected inactivated polio vaccine (IPV) may induce nasal and pharyngeal mucosal immunity, we measured PV type-specific neutralization and immunoglobulin (Ig) G, IgA, and IgM levels in nasal secretions, adenoid cell supernatants, and sera collected from 12 children, aged 2–5 years, undergoing planned adenoidectomies. All participants were routinely immunized with IPV and had no known contact with live PVs.

**Results:**

PV-specific mucosal neutralization was detected in nasal and adenoid samples, mostly from children who had previously received 4 IPV doses. Across the 3 PV serotypes, both nasal (Spearman ρ ≥ 0.87, *P* ≤ .0003 for all) and adenoid (Spearman ρ ≥ 0.57, *P* ≤ .05 for all) neutralization titers correlated with serum neutralization titers. In this small study sample, there was insufficient evidence to determine which Ig isotype(s) was correlated with neutralization.

**Conclusions:**

Our findings provide policy-relevant evidence that routine immunization with IPV may induce nasal and pharyngeal mucosal immunity. The observed correlations of nasal and pharyngeal mucosal neutralization with serum neutralization contrast with previous observations of distinct intestinal and serum responses to PV vaccines. Further research is warranted to determine which antibody isotype(s) correlate with polio vaccine–induced nasal and pharyngeal mucosal neutralizing activity and to understand the differences from intestinal mucosal immunity.

## Teaser

This study provides policy-relevant evidence that children routinely immunized with inactivated polio vaccine in the United States and who had no known contact with a live poliovirus had detectable nasal and pharyngeal mucosal immunity to poliovirus.

Polioviruses (PVs) are single-stranded positive-sense RNA enteroviruses comprising 3 serotypes (PV1, PV2, and PV3) [1]. PVs are transmitted via oral-oral and fecal-oral routes and replicate in lymphoid tissue of the pharynx (ie, adenoids and tonsils) and the ileum (ie, Peyer's patches) [2, 3]. Among immunocompetent individuals, PV shedding after infection can commonly be detected for 1–2 weeks in the pharyngeal secretions and 3–6 weeks in the stools (reviewed in [1]).

Existing research on mucosal immunity to PVs has predominantly focused on intestinal immunity, measured in stool samples as PV type-specific neutralizing activity and immunoglobulin (Ig) A (reviewed in [4, 5]). A series of studies in which previously vaccinated participants were challenged with a live attenuated oral polio vaccine (OPV) demonstrate that PV shedding in stool can be effectively inhibited by prior receipt of a homologous OPV, but not by prior receipt of a homologous inactivated polio vaccine (IPV) administered intramuscularly [5–8]. Accumulating evidence also suggests that active viral replication in the intestinal tract is necessary to induce both intestinal mucosal neutralization and PV-specific IgA responses [7, 9, 10]. Further, enhancing serum neutralization by increasing the number [8] or potency [7, 11] of IPV doses in infants appears to have little effect on intestinal viral excretion and intestinal neutralizing antibody responses upon subsequent exposure to live vaccine virus.

However, little is known about the induction of nasal and pharyngeal mucosal immunity after polio vaccination [5, 12]. There is evidence of polio elimination using IPV-only schedules in some countries with high levels of sanitation and vaccination coverage (eg, Sweden, Iceland, Finland, and The Netherlands) [13]. As IPV is known to have little impact on fecal shedding, it has been hypothesized that the elimination of polio within these countries likely reflects the reduction of oral-oral transmission due to the induction of pharyngeal mucosal immunity [14, 15]. Supporting this hypothesis, a vaccine challenge trial conducted from 1980 to 1983 using type 1 OPV (OPV1) reported detecting OPV1 shedding in 1% (1/93) of pharyngeal samples and 63% (59/93) of stool samples from children previously vaccinated with IPV and 4% (3/79) of pharyngeal samples and 25% (20/79) of stool samples from children previously vaccinated with trivalent OPV [16].

The recent detection of circulating vaccine-derived PVs in previously polio-free countries that have IPV-only routine immunization schedules has renewed interest in the ability of IPV to induce pharyngeal mucosal immunity and reduce oral-oral transmission [12]. In October 2022, the World Health Organization Strategic Advisory Group of Experts recommended the use of IPV in response to geographically limited polio outbreaks in countries with IPV-only vaccination schedules and high levels of sanitation and surveillance [17].

To investigate the extent to which routine immunization with intramuscularly injected IPV may induce nasal and pharyngeal mucosal immunity, we measured PV serotype-specific neutralization and IgG, IgA, and IgM binding antibodies in nasal secretions (ie, samples from naso-absorption sponges and nasal washes), adenoid cell supernatants, and sera collected from children undergoing planned adenoidectomies in the United States (US). We examined the pairwise correlations in neutralization across sample types. Within each sample type, we described PV serotype-specific neutralization and IgG, IgA, and IgM overall and in relation to the number of IPV doses previously received and the duration of time since the last IPV dose and investigated the correlations between neutralizing activity and IgG, IgA, and IgM levels.

## MATERIALS AND METHODS

### Study Design

This evaluation of PV-specific mucosal and serum immunity in IPV-vaccinated children used samples that were collected as part of a broader investigation of the development of mucosal immunity to severe acute respiratory syndrome coronavirus 2 (SARS-CoV-2). The clinical study took place between 8 July and 16 November 2022 at Dartmouth-Hitchcock Medical Center (Lebanon, New Hampshire). Eligible participants included children <5 years of age, undergoing a planned adenoidectomy or adenotonsillectomy for clinical indications (eg, Eustachian tube dysfunction, sleep-disordered breathing, and/or recurrent pharyngitis). All children were otherwise healthy with no known immunodeficiency. All but 1 child had documentation of a prior SARS-CoV-2 infection. All participants had been routinely immunized with IPV (the routine pediatric immunization schedule in the US included IPV at 2, 4, and 6–18 months with a booster at 4–6 years [18]) and had not traveled outside of the US.

### Sample Collection

After intubation of the patient under general anesthesia, blood samples were obtained. At the start of the surgical procedure, nasal secretions from the child's anterior nares were collected for 1 minute using a naso-absorption sponge (Nasosorption FX-i, Hunt Developments Ltd, Midhurst, United Kingdom) followed by a 2-mL nasal wash with normal saline collected with a Lukens trap. The adenoid specimen was removed with an adenoid curette and placed in sterile saline. All samples were held at room temperature in the operation room for ≤30 minutes after collection. The adenoid tissues were cleaned of fat and debris and homogenized on a scored plate in tissue media (RPMI with 5% fetal bovine serum and 0.24% HEPES). Plates were washed, and dispersed tissues were filtered through a 70-µm cell strainer. The resulting adenoid tissue filtrate was spun into pellet cells, and supernatant was stored for immunological investigation. Of note, the adenoid cell supernatant may have been contaminated by blood arising from adenoid vascularization or during the surgical procedure. The volume of diluent fluid used was 0.5 mL for nasal sponge, 2 mL for nasal wash, and 20–30 mL for the adenoid supernatant based on the size of the tissue. All samples were de-identified, aliquoted, and stored frozen at −40°C at Dartmouth-Hitchcock Medical Center.

### Laboratory Procedures

PV1-, PV2-, and PV3-specific neutralizing activity in nasal sponge samples, nasal washes, adenoid cell supernatant, and serum were assayed and results were reported as the reciprocal of the highest sample dilution that achieved 60% neutralization of a luciferase-expressing polio pseudovirus [9, 19]. Undetectable titers (ie, <1:4 dilution) were recorded as 2, and titers higher than the upper limit of detection (ie, >1:512 dilution) were recorded as 1024. Total Ig concentrations (µg/mL) and PV1-, PV2-, and PV3-specific IgG, IgA, and IgM levels in nasal sponge samples, nasal washes, adenoid cell supernatant, and serum were quantified using a multiplex assay developed by coupling monovalent IPV to fluorescently coded magnetic microspheres, as described previously [8, 9]. PV-specific Ig levels were reported as log_10_ mean fluorescence intensities (MFIs). Sample measurements that did not exceed 10 standard deviations (SD) above buffer blanks were set to this value. To note, PV2-specific IgG in serum was not determined due to technical issues that resulted in an abnormally high blank MFI value.

### Statistical Analysis

PV1-, PV2-, and PV3-specific log_2_ neutralization titers and proportions of participants with detectable virus-neutralizing activity (ie, log_2_ neutralization titers >1), as well as total IgG, IgA, and IgM concentrations, were reported for each sample type (ie, nasal sponge samples, nasal washes, adenoid cell supernatants, and serum). Correlations were estimated using Spearman rank correlation coefficients and visualized using scatter plots, comparing (*i*) PV serotype-specific neutralization titers across the different sample types; (*ii*) PV serotype-specific neutralization titers and IgG, IgA, and IgM MFIs within each sample type; and (*iii*) PV serotype-specific neutralization titers and IgG, IgA, and IgM MFIs with the time (in days) since the last IPV vaccination in each sample type. The distribution of PV-specific neutralization titers in nasal sponge samples, adenoid cell supernatant, and serum stratified by the number of IPV doses received were plotted. As nasal washes were not available for all participants, they were excluded from the main analysis. As a sensitivity analysis for the nasal collection method, the correlations between PV-specific neutralization titers in the nasal washes (ie, the conventional sampling method) and in the nasal sponge samples and between the PV-specific IgG MFIs from both nasal sampling methods were estimated using Spearman rank correlation coefficients and visualized using scatterplots.

All *P* values are from 2-sided statistical tests. All analyses were performed using Stata version 17.0 and R version 4.2.0 software.

### Ethics

The primary study protocol was approved by the Dartmouth-Hitchcock Institutional Review Board (#02001499). Informed consent was provided by a single parent or guardian. Further consent from the parent/guardian to use the samples for the PV study was obtained along with information on the polio vaccination history of the children. Samples were de-identified prior to undertaking immunoassays and analyses.

## RESULTS

PV serotype-specific mucosal and serum immune neutralizing activity and binding antibodies, as well as total IgG, IgA, and IgM, were evaluated in 12 children (age range, 2–5 years; mean age, 3.4 [SD, 1.1] years) of whom 8 (67%) were female. Seven children (58%; age range, 3–5 years) had received 4 IPV vaccinations; of these, 2 received the fourth dose early at 1 year of age, and 5 received it at the recommended 4 years of age. Four children (33%; age range, 2–3 years) had received 3 doses of IPV, with the third dose at the age of 6 months, and were too young to have received the fourth dose. One 2-year-old child had only received 2 of the 3 IPV doses recommended for their age.

**Figure 1.**
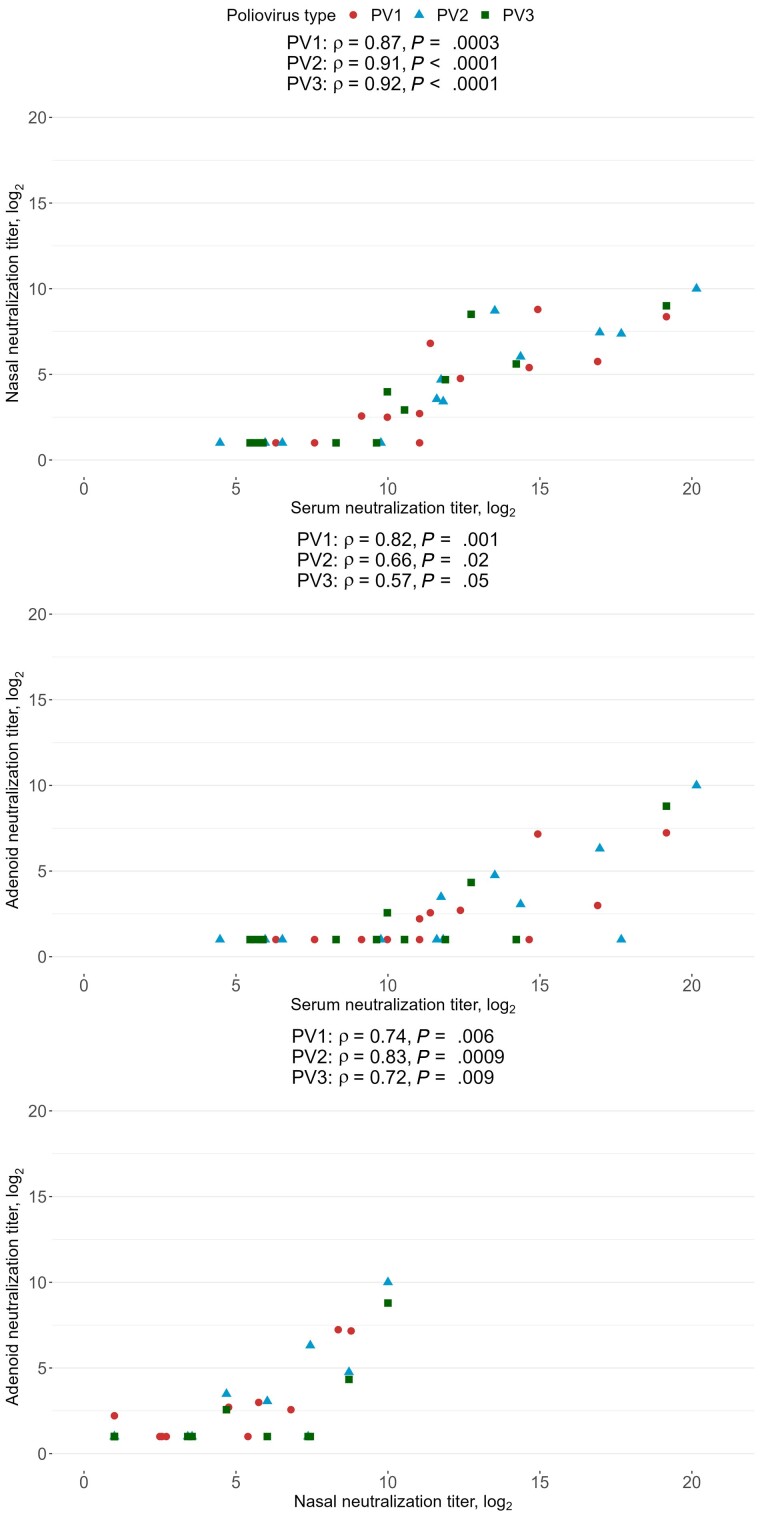
Correlations between poliovirus (PV) type-specific neutralization titers in nasal sponge samples, adenoid cell supernatants, and serum. Correlation coefficients are estimated from Spearman rank correlations. Undetectable log_2_ neutralization titers were recorded as 1. Red circles, PV1; blue triangles, PV2; green squares, PV3.

Overall, neutralizing activity in nasal sponge samples was detected (ie, log_2_ neutralization titers >1) in 9 of 12 (75%) participants against PV1, 8 of 12 (67%) participants against PV2, and 6 of 12 (50%) participants against PV3 (Table 1). Three children, who had each received 2 or 3 IPV doses, had no detectable nasal neutralizing activity against any of the 3 PV serotypes. In adenoid cell supernatant, neutralizing activity was detected in 6 of 12 (50%) participants against PV1, 5 of 12 (42%) participants against PV2, and 3 of 12 (25%) participants against PV3. In serum samples, neutralizing activity was detected in all participants for all 3 PV serotypes (Table 1). For each PV serotype, the neutralization titers in nasal sponge samples and serum were strongly and positively correlated (Spearman ρ = 0.87, *P* = .0003 for PV1; ρ = 0.91, *P* < .0001 for PV2; ρ = 0.92, *P* < .0001 for PV3; Figure 1). Similarly, the neutralization titers in adenoid samples and serum were positively correlated (Spearman ρ = 0.82, *P* = .001 for PV1; ρ = 0.66, *P* = .02 for PV2; ρ = 0.57, *P* = .05 for PV3). Neutralization titers were also strongly correlated between nasal and adenoid samples (Figure 1).

**Table 1.** Poliovirus Type-Specific Mucosal and Serum Neutralizing Activity and Total Immunoglobulin (Ig) G, IgA, and IgM Concentrations in 12 Children Previously Vaccinated With Inactivated Polio Vaccine

Poliovirus Type	Nasal Sponge (n = 12)	Nasal Washes (n = 12)^a^	Adenoid (n = 12)	Serum (n = 12)
PV1				
Detectable neutralizing activity	9/12 (75.0%)	7/9 (77.8%)	6/12 (50.0%)	12/12 (100%)
Neutralization titers, log_2_	3.7 (1.7–6.3)	3.4 (2.4–3.7)	1.6 (1–2.8)	11.2 (9.6–14.8)
PV2				
Detectable neutralizing activity	8/12 (66.7%)	10/12 (83.3%)	5/12 (41.7%)	12/12 (100%)
Neutralization titers, log_2_	4.1 (1–7.4)	3.4 (2.2–5.2)	1 (1–4.1)	11.8 (8.1–15.7)
PV3				
Detectable neutralizing activity	6/12 (50.0%)	4/7 (57.1%)	3/12 (25.0%)	12/12 (100%)
Neutralization titers, log_2_	2.0 (1–5.1)	2.1 (1–4.2)	1 (1–1.8)	9.8 (5.9–12.3)
Total				
IgG, µg/mL	819 (560–1119)	820 (23–994)	80 (40–424)	7561 (6030–10 437)
IgA, µg/mL	765 (423–1311)	617 (13–1659)	11 (4–22)	1434 (889–1885)
IgM, µg/mL	188 (163–365)	262 (207–445)	2 (1–5)	10 284 (6504–11 893)

Data are presented as median (interquartile range) or no./No. (%). Log_2_ neutralization titers >1 were considered as detectable.

Abbreviations: IgA, immunoglobulin A; IgG, immunoglobulin G; IgM, immunoglobulin M; PV1, poliovirus type 1; PV2, poliovirus type 2; PV3, poliovirus type 3.

^a^n = 9 for PV1 and n = 7 for PV3.

**Figure 2.**
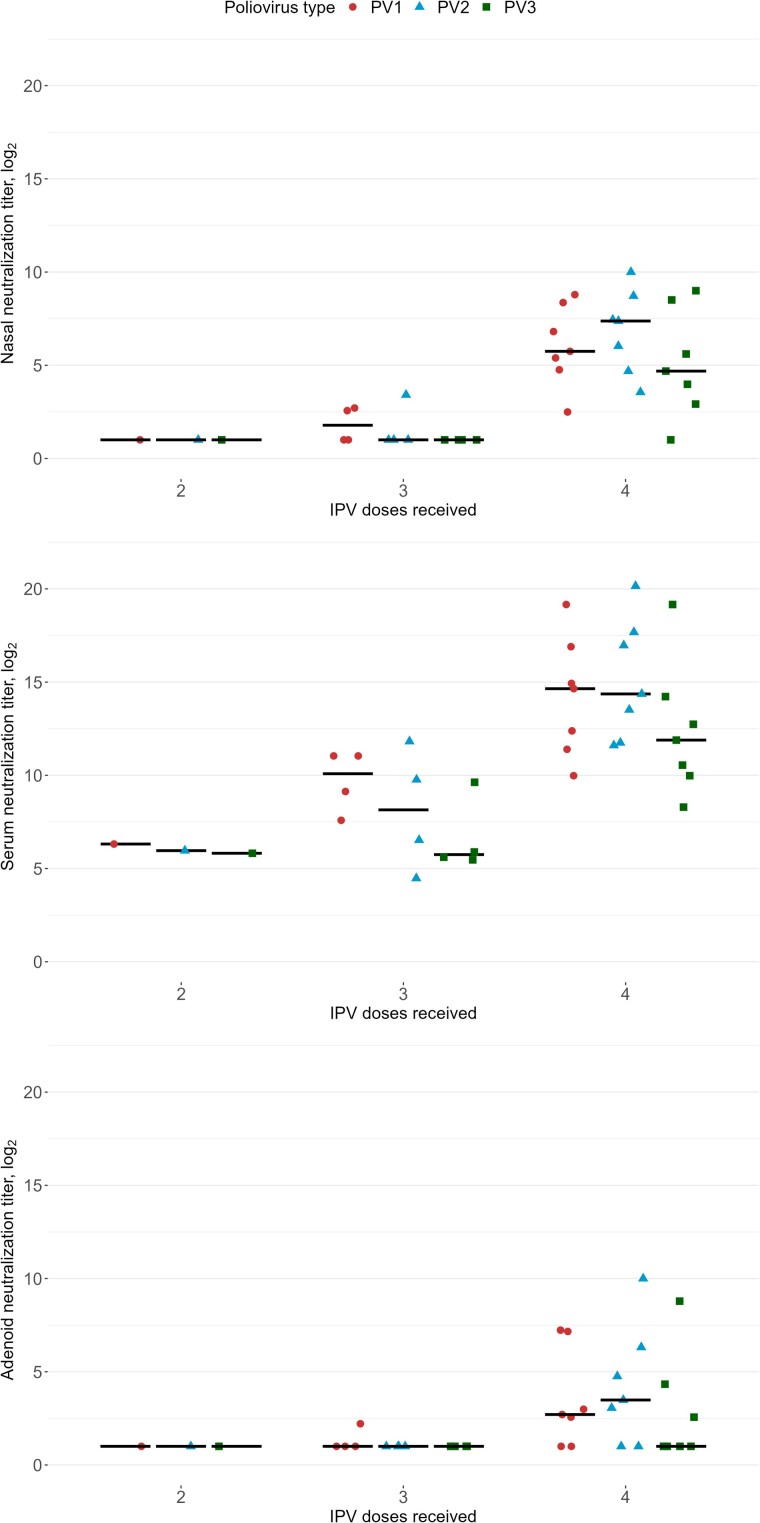
Distribution of poliovirus (PV) type-specific neutralization titers in nasal sponge samples, adenoid cell supernatants, and serum, stratified by the number of inactivated polio vaccine (IPV) doses previously received. Undetectable log_2_ neutralization titers were recorded as 1. Red circles, PV1; blue triangles, PV2; green squares, PV3. Horizontal bars indicate the median levels. Children per dose of IPV received: 2 doses (n = 1), 3 doses (n = 4), 4 doses (n = 7).

**Figure 3.**
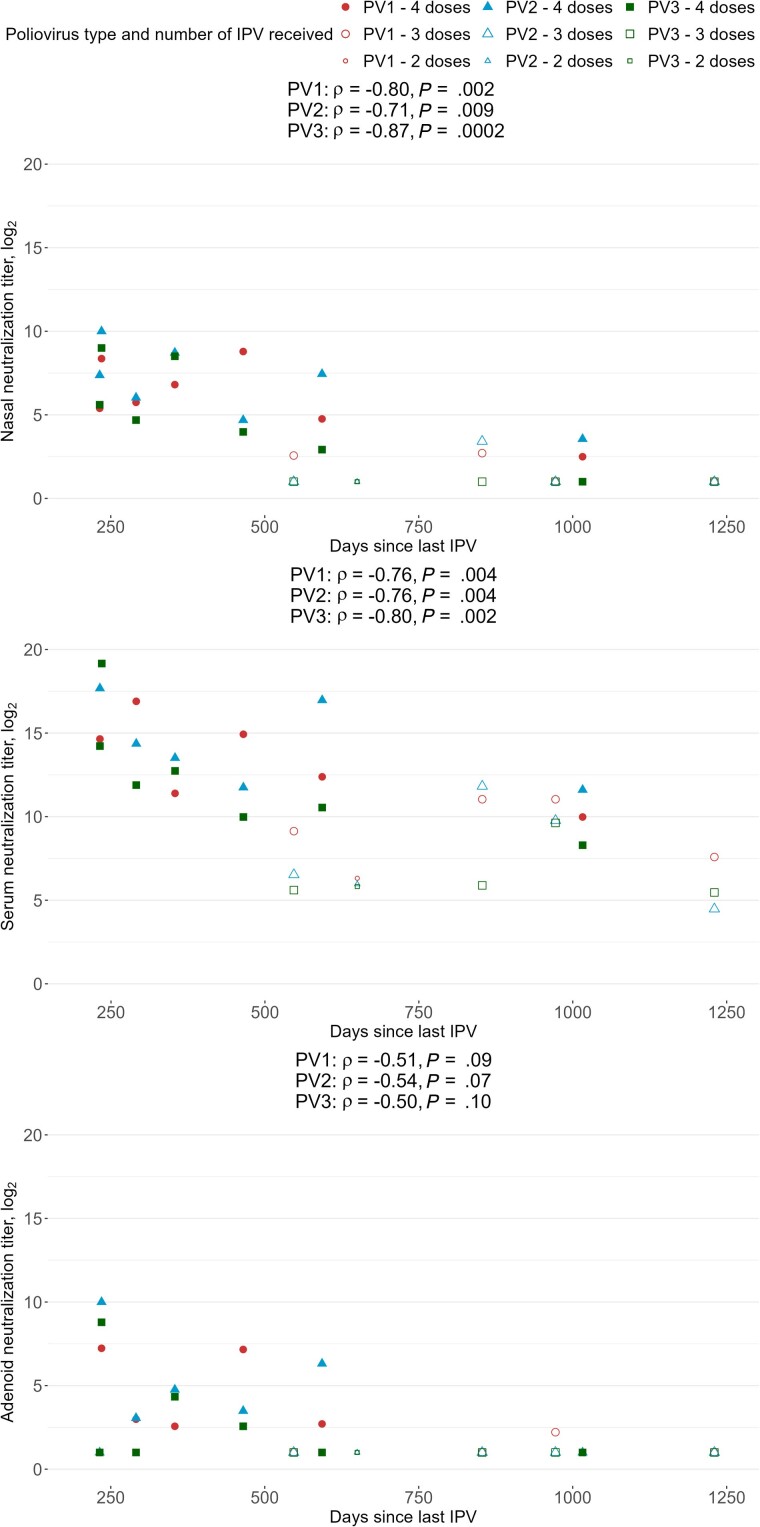
Correlations between poliovirus (PV) type-specific neutralization titers from nasal sponge samples, adenoid cell supernatants, and serum and the time since the last dose of inactivated polio vaccine (IPV) received (in days). Correlation coefficients are estimated from Spearman rank correlations. Undetectable log_2_ neutralization titers were recorded as 1. Red circles, PV1, blue triangles, PV2, and green squares, PV3. Large shaded symbols, 4 doses of IPV; large open symbols, 3 doses of IPV; small open symbols, 2 doses of IPV.

In the sera, neutralization titers were higher among the children who had received 4 doses of IPV compared to those who had 3 doses (Figure 2). In the nasal sponge and adenoid samples, neutralizing activity was mostly detected in children who had received 4 doses of IPV (Figure 2). All the children who had received 4 IPV doses had detectable neutralizing activity for all 3 PV serotypes in the nasal sponge samples, except for 1 child whose PV3-specific neutralizing activity was undetectable (n.b., this child had received their fourth dose at 1 year of age) (Figure 2). Of the 4 children who had received 3 IPV doses, 2 had no detectable neutralization in the nasal samples against any PV serotypes, while 1 had PV1- and PV2-specific detectable neutralization, and 1 had PV1-specific detectable neutralization. However, it should be noted that the children who had received 3 doses of IPV also had a longer duration of time between the last vaccination and the sample collection (ie, mean 900 days) than those who had received 4 doses (ie, mean of 455 days) (*P* = .03, *t* test). The child who received 2 doses, the last one 650 days before the sampling, had no detectable neutralizing activity in the nasal sponge sample and adenoid cell supernatant. We observed a decay in neutralization titers that correlated with the time since the last dose of IPV received (Figure 3). Consistently among all 3 PV serotypes, neutralization titers from the nasal sponge, adenoid, and serum samples were strongly inversely correlated with the time since the last IPV dose (Figure 3). The PV serotype-specific IgG, IgA, and IgM MFIs and the time since the last dose of IPV received were not significantly correlated (Supplementary Figure 1).

PV-specific IgG and IgM were detected in all sample types, whereas PV-specific IgA was only detected in nasal sponge samples and serum. The correlations between PV-specific IgG and neutralization titers were positive but not statistically significant in nasal sponge samples (Spearman ρ ≥ 0.10, *P* ≥ .19 for all serotypes) and serum samples (Spearman ρ ≥ 0.43 and *P* ≥ .07 for PV1 and PV3; Supplementary Figure 2). No significant correlations were observed between PV-specific nasal sponge neutralization and serum IgG levels (Spearman ρ = 0.34, *P* = .27 for PV1; ρ = 0.49, *P* = .11 for PV2; ρ = 0.25, *P* = .44 for PV3). Furthermore, PV-specific IgA and neutralization titers were not correlated in the nasal sponge samples (*P* ≥ .54 for all serotypes, Supplementary Figure 2). In the adenoid cell suspension, a positive correlation of the neutralization titers with IgG and IgM was observed (Supplementary Figure 2); however, only ≤50% of samples had both detectable neutralization and PV-specific IgG or IgM.

As a sensitivity analysis for the nasal collection method, we compared the PV-specific neutralization titers and IgG, IgA, and IgM levels in nasal secretions obtained by nasal sponges versus the more traditional nasal washes. We observed a correlation between the neutralization titers in nasal sponge samples and nasal washes only for data on the PV2 serotype, for which we had no missing samples (Supplementary Figure 3). We also observed a positive correlation of the PV-specific IgG levels (Spearman ρ ≥ 0.63) between the 2 collection methods (Supplementary Figure 3). Moreover, total IgG, IgA, and IgM concentrations were similar between the nasal washes and the nasal sponge samples, but caution is warranted in the interpretation as the nasal washes were more diluted than the nasal sponge samples (Table 1).

## DISCUSSION

In this study, we evaluated the extent of nasal and pharyngeal mucosal immunity to PV induced by vaccination using nasal secretions, adenoid cell supernatant, and serum from 12 IPV-vaccinated children. In nasal sponge and adenoid samples, we observed neutralizing activity mostly in children who had received 4 doses of IPV, whereas we detected serum neutralizing activity in all children. We observed a strong correlation between neutralizing activity in the nasal or pharyngeal mucosae (ie, both nasal and adenoid samples) and in the serum. Detection of neutralizing activity in mucosal samples from children previously vaccinated with IPV who had no known contact with a live virus (ie, OPV or circulating PV), and its strong correlation with serum neutralizing activity, provides evidence that vaccination with IPV induced nasal and pharyngeal mucosal immunity, possibly derived from serum antibodies.

Our results suggest that the induction of nasopharyngeal mucosal immunity to PV differs from intestinal mucosal immunity, as previous studies have reported low but detectable PV2-specific stool neutralization (ie, 26%–37% of detectable neutralization before challenge by a live vaccine) in infants immunized by IPV only or bivalent OPV with IPV schedules [6, 8]. In studies assessing nasopharyngeal mucosal immunity after vaccination in infants, neutralizing activity in nasal samples was more likely to be detected after OPV (ie, detected in approximately 70% of infants after 2 doses and 70%–100% after 3 doses) than IPV (ie, detected in approximately 30% of infants after 2 doses and 43%–90% after 3 doses) [20, 21]. In 2 earlier studies of viral excretion among household contacts of paralytic cases, PV excretion in the pharynx, collected by pharyngeal swab, was observed less frequently in IPV-vaccinated compared to nonvaccinated contact children [22], and reduced pharyngeal shedding was associated with higher serum antibody titers in IPV-vaccinated contact children [14].

Mucosal neutralization titers were mostly observed in children who had received 4 doses of IPV in our study, but this finding may be confounded by the time between the measurement and the last vaccination. The persistence of antibody responses in the nasopharynx has been documented in previous studies showing stable IgA levels for up to 6 years after OPV vaccination [23]. Our results show that nasal neutralizing activity can persist up to 2.8 years after IPV vaccination in children who had received the full primary immunization series. More research is needed to assess the duration of nasopharyngeal mucosal immunity, the independent effect of the number of vaccine doses received, and the relation between nasopharyngeal mucosal immunity and viral excretion. Of note, we observed the presence of PV-specific IgA in the nasal samples and sera. Although a modest increase of secretory IgA in the nasopharynx after a booster by IPV in IPV-primed children has been reported [23], other previous studies have reported absent or low IgA responses that were considered to be dependent on prior immunization and exposure history [24–27]. Further research is needed to determine whether these discrepancies result from different assay protocols and/or sensitivities, the relatively permissive threshold selected for detection of responses used here (ie, to support the investigation of relationships with neutralization activity), the potential cross-reactivity of antibodies to other enteroviruses, or other factors.

The observed correlation between the serum and the nasal and pharyngeal mucosal neutralization titers suggests that immunity in the pharynx may be gained through the serum after IPV vaccination. In previous studies assessing IPV-induced immunity to PVs, the induction of neutralizing and binding antibody responses in serum and stool samples appeared to be independent, such that serotype-specific serum neutralization titers were not associated with levels of serotype-specific stool neutralization, IgA levels, or stool viral shedding [7, 8]. Accumulating evidence suggests that intestinal mucosal neutralization is mostly mediated by IgA [4, 7]. In our study, there was insufficient evidence to conclude that nasal neutralization was mediated by any specific Ig isotype measured. PV-specific IgG and IgM were not detected in nasal wash in earlier studies in the 1960s, while IgA was [10, 28]. However, low-level PV-specific IgG in nasal wash has been found temporarily in children after adenotonsillectomy and was attributed to exudation due to mucosal inflammation after surgery [28]. Further investigation measuring mucosal immunity in the nasopharynx concomitantly to the stools in infants and children with different vaccine backgrounds is necessary to understand how the mucosal immunity develops and which antibody type(s) may be the determinant of polio mucosal immunity across body sites.

The primary nasal sampling collection method, the nasal sponge, was newly employed to collect nasal secretions. We observed a correlation between the PV2-specific neutralization titers from the nasal sponge samples and the nasal washes. Similarly, while not significant due to the small sampling size, we observed a positive correlation of the PV-specific IgG levels between the 2 collection methods. While our analysis was limited by the lack of some nasal wash samples and the difference in the volume of diluent (ie, a 4-times higher volume in the washes than in the sponge samples), these results are encouraging and suggest that the 2 methods are likely similar. The nasal sponge procedure is not traumatic and specifically samples the anterior nasal cavity, making it unlikely to be from serum leakage, as may be the case with saliva. We postulate that it may result in a better sampling of the mucociliary blanket and the adjacent cellular domains. Additionally, sponge samples are collected in a smaller volume than the nasal wash and other mucosal sampling techniques, resulting in less need to concentrate the samples after collection.

Our study provided an opportunity to study nasal and pharyngeal mucosal immunity in children who were most likely unexposed to live PVs, as the children had not traveled outside of the US, and there was no known PV circulation in the US during the study participants' lives (wild PVs were eliminated in the US in 1979 and OPV has not been used since 2000 [29]). This study also provided an opportunity to assess a new collection method in the nasal sponge, which resulted in similar neutralization and binding antibody patterns as compared to the nasal wash method. Further, this study enabled us to assess adenoid samples concomitantly with nasal samples, but caution is warranted in the interpretation of the adenoid cell supernatant results as they may have been contaminated by blood, and as the samples were highly diluted. While the consistency in our results (ie, the strong correlation of neutralization titers between nasal sponges and adenoid samples in all 3 PV serotypes) likely reflects locally produced adenoid antibodies or possibly a transfer from serum in both compartments, more research is needed to identify which antibody isotype(s) are associated with the observed PV neutralizing activity. The main limitation of this study was the small size sample, limiting the ability to detect small effects and the generalizability to other age groups, other settings, and different vaccine backgrounds (eg, IPV associated with bivalent OPV or Sabin IPV vaccination). Second, the analysis was limited by the absence of longitudinal measures in nasal samples to describe the kinetics of the immune response, as well as the absence of concomitant stool measures needed to support comparison with existing literature.

Since polio outbreak responses may include the use of IPV in countries using IPV exclusively or with persistent circulation despite multiple OPV campaigns, understanding how IPV vaccination could limit PV transmission is key to the polio endgame. Further research will be invaluable to understand if the induction of nasopharyngeal and intestinal mucosal immunity is indeed separate, how nasopharyngeal neutralization is related to viral excretion, and the extent to which nasopharyngeal mucosal immunity may contribute to the reduction of poliovirus transmission.

## Supplementary Data

Supplementary materials are available at *The Journal of Infectious Diseases* online (http://jid.oxfordjournals.org/). Supplementary materials consist of data provided by the author that are published to benefit the reader. The posted materials are not copyedited. The contents of all supplementary data are the sole responsibility of the authors. Questions or messages regarding errors should be addressed to the author.

## Supplementary Material

jiae264_Supplementary_Data

## Notes

***Acknowledgments***. This study was conducted as part of the Dartmouth International Vaccine Initiative.

***Author contributions*.** A. G.: methodology, validation, formal analysis, investigation, data curation, writing—original draft, writing—review & editing, visualization. R. I. C.: conceptualization, methodology, validation, investigation, writing—review & editing. H. N. D., K. S. A., and G. K.: investigation, writing—review & editing. P. C. R.: conceptualization, investigation, writing—review & editing, supervision, project administration, funding acquisition. W. F. W.-A.: methodology, validation, investigation, data curation, writing—review & editing, project administration. J. A. W.: methodology, validation, investigation, data curation, writing—review & editing. M. E. A.: conceptualization, investigation, resources, writing—review & editing, supervision, project administration, funding acquisition. E. Y. C.: conceptualization, investigation, writing—review & editing, funding acquisition. M. A.: resources, writing—review & editing. A. S. B. and A. I. R.: writing—review & editing. J. M.: conceptualization, methodology, investigation, writing—review & editing. E. B. B.: conceptualization, methodology, investigation, formal analysis, resources, writing—original draft, writing—review & editing, supervision, project administration. P. F. W.: conceptualization, methodology, investigation, resources, writing—original draft, writing—review & editing, supervision, project administration, funding acquisition.

***Financial support***. This work was supported by the Bill & Melinda Gates Foundation (grant number GC10058 to P. F. W.); Geisel School of Medicine at Dartmouth College (Munck-Pfefferkorn Grant Award to P. C. R.); Dartmouth bioMT through the National Institutes of Health/National Institute of General Medical Sciences (grant number P20-GM113132 to P. C. R.); and Japan Agency for Medical Research and Development (grant number AMED 24fk0108627j0003 to M. A.).
