# Supplementary material for: Nasal and Pharyngeal Mucosal Immunity to Poliovirus in Children Following Routine Immunization With Inactivated Polio Vaccine in the United States
Source: J Infect Dis. 2024 May 29;230(5):e1023–30. doi: 10.1093/infdis/jiae264 (PMC11566227; doi:10.1093/infdis/jiae264)
Supplement: jiae264_Supplementary_Data [file jiae264_supplementary_data.docx]

Supplementary Figure 1. Correlations between poliovirus (PV) type-specific immunoglobulin (Ig)G, IgM, and IgA levels from nasal sponge samples, adenoid cell supernatants, and serum, and the time since the last dose of inactivated polio vaccine (IPV) received (in days). Correlation coefficients are estimated from Spearman’s rank correlations. Red circles = PV1, blue triangles = PV2, and green squares = PV3; large shaded symbols = 4 doses of IPV, large open symbols = 3 doses of IPV, and small open symbols = 2 doses of IPV. Igs mean fluorescence intensities (MFIs) levels lower than the lower limit of detection were excluded from these analyses. To note, serum PV2 IgG was not determined due to technical issues that resulted in an abnormally high buffer blank MFI value, and IgA MFIs were not detectable in the adenoid samples.

Supplementary Figure 2. Correlations of poliovirus (PV) serotype-specific neutralization titers and (A) immunoglobulin (Ig) G, (B) IgA, and (C) IgM mean fluorescence intensities (MFIs) from the nasal sponge samples, adenoid cell supernatants, and serum. Correlation coefficients are estimated from Spearman’s rank correlations. Undetectable log_2_ neutralization titers were recorded as 1. Red circles = PV1, blue triangles = PV2, and green squares = PV3; shaded symbols = detectable Ig, and open symbols = undetectable Ig. To note, serum PV2 IgG was not determined due to technical issues that resulted in an abnormally high buffer blank MFI value, and IgA MFIs were not detectable in the adenoid samples. Children included in the analyses: n=12.


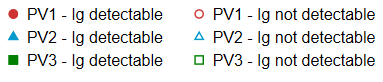

Supplementary Figure 3. Correlations of poliovirus (PV) serotype-specific (A) neutralization titers and (B) immunoglobulin (Ig) G mean fluorescence intensities (MFIs) between nasal sponge and nasal wash samples. Correlation coefficients are estimated from Spearman’s rank correlations. Undetectable log_2_ neutralization titers were recorded as 1. Red circles = PV1, blue triangles = PV2, and green squares = PV3. Samples available for the neutralization assay: n=9 for PV1, n=12 for PV2, and n=7 for PV3. Samples available for the measurement of IgG MFIs measure: n=8 for all PV serotypes.
